# What happens to people admitted to a specialist dementia unit in the west of Scotland?

**DOI:** 10.1192/bjo.2021.840

**Published:** 2021-06-18

**Authors:** Andrew Donaldson, Craig Patrick, Lindsay Short, Helen Maginnis

**Affiliations:** NHS Lanarkshire

## Abstract

**Aims:**

Recent local research examined mortality rates following admission to a dementia ward. We wanted to expand on this work and include other important health outcomes for patients admitted to our specialist in-patient dementia unit in the west of Scotland. This would provide a comprehensive overview of our in-patient population, aid service review and improve care. We hypothesised that patients admitted would be physically frail, have a significant mortality rate and would likely require long-term care post discharge.

**Method:**

The clinical notes for each admission to the unit for one year were examined (total 62). We extracted data from a number of different areas such as demographics, mortality rates, discharge destination, readmission rates and prescribed medications.

**Result:**

60% had an Alzheimer's/mixed dementia diagnosis. Average length of stay was 64 days. 62% were discharged to a care home (50% of this total had lived at home prior to admission), 18% to complex care and 20% to the community. 66% were prescribed an antipsychotic and the average number of medications was 8.4. 35% had a readmission under general medicine within a year of discharge. 19% died whilst an inpatient and a further 30% had died one year post-discharge (total one-year mortality of 44%).

**Conclusion:**

People admitted to our dementia unit are physically frail, with only 20% returning to live in the community, 35% being readmitted to a general medical ward within a year of discharge and 44% dying during the admission or within a year of discharge. We need to bear these results in mind when considering if hospital admission is appropriate and ultimately further develop our skills in palliative and end of life care in order to provide those people admitted to our dementia unit (and those who remain at home) with the highest standard of care.

